# Mediterranean diet, folic acid, and neural tube defects

**DOI:** 10.1186/s13052-017-0391-7

**Published:** 2017-08-17

**Authors:** Maximilian Fischer, Mauro Stronati, Marcello Lanari

**Affiliations:** 1 Department of Pediatric Emergency, S. Orsola-Malpighi Hospital, University of Bologna, Bologna, Italy; 2 Neonatal Intensive Care Unit, IRCCS Foundation Policlinico San Matteo, Pavia, Italy

**Keywords:** Mediterranean diet, Pregnancy, Neural tube defects, Congenital defects, Folic acid, Folate, Fortification, Supplementation

## Abstract

The Mediterranean diet has been for a very long time the basis of food habits all over the countries of the Mediterranean basin, originally founded on rural models and low consumption of meat products and high-fat/high-processed foods. However, in the modern era, the traditional Mediterranean diet pattern is now progressively eroding due to the widespread dissemination of the Western-type economy, life-style, technology-driven culture, as well as the globalisation of food production, availability and consumption, with consequent homogenisation of food culture and behaviours. This transition process may affect many situations, including pregnancy and offspring’s health. The problem of the diet during pregnancy and the proper intake of nutrients are nowadays a very current topic, arousing much debate. The Mediterranean dietary pattern, in particular, has been associated with the highest risk reduction of major congenital anomalies, like the heterogeneous class of neural tube defects (NTDs). NTDs constitute a major health burden (0.5-2/1000 pregnancies worldwide) and still remain a preventable cause of still birth, neonatal and infant death, or significant lifelong disabilities. Many studies support the finding that appropriate folate levels during pregnancy may confer protection against these diseases. In 1991 one randomised controlled trial (RCT) demonstrated for the first time that periconceptional supplementation of folic acid is able to prevent the recurrence of NTDs, finding confirmed by many other subsequent studies. Anyway, the high rate of unplanned/unintended pregnancies and births and other issues hindering the achievement of adequate folate levels in women in childbearing age, induced the US government and many other countries to institute mandatory food fortification with folic acid. The actual strategy adopted by European Countries (including Italy) suggests that women take 0,4 mg folic acid/die before conception. The main question is which intervention, between folic acid supplementation, foods fortification or both, linked to a healthy life-style and diet pattern may represent the best method in preventing NTDs. The aim of this review is to describe the actual situation in NTDs prevention, with a special attention to the Italiancontext concerning this delicate and controversial subject.

## Background

### Mediterranean diet, a senseless decline

Nutritional epidemiology studies have risen during last recent years, mostly stimulated by the increasing evidence of the primary role of eating habits on health status [1].

In this context, many observational studies are reporting how a diet following the basic principles of the traditional Mediterranean dietary pattern is strictly associated with a better health status and has been considered a standard of nutritional quality due to its main components [2]. The origins of the “Mediterranean Diet” are very lost in time as they mainly sink into ancient Greek and Roman eating traditions, based on the use of cereals, wine and olives products, known as “the Mediterranean triad”, and supplemented by sheep cheese, vegetables (leeks, mallow, lettuce, chicory, mushrooms), little meat and a strong propensity for fish and seafood [3].

Up to now, there is no univocal definition of this specific dietary pattern. Nevertheless, as mentioned above, there are some common features that, in fact, typify the dietary pattern and benefits of the Mediterranean diet: among the most important, plant-derived foods (fruits, vegetables, whole-grain cereals, legumes, and red wine in moderation) provide a variety of natural antioxidants (i.e. vitamins and phenolic compounds), dietary fiber, and resistant starches; olive oil, nuts, and fish, as the main source of fat, provide mono- and polyunsaturated long chain fatty acids (MUFA and PUFA). These dietary habits are associated with a traditional low consumption of meat-products and high-fat high-processed foods, which in turn have been associated with adverse effects on health (e.g. obesity, hypertension, coronary artery disease, stroke, peripheral artery disease, diabetes, etc) [4].

The discovery of the health benefits of the Mediterranean Diet dates back to the 50s and is attributed to the american scientist Ancel Keys (University of Minnesota), who pointed out the correlation between cardiovascular disease, lifestyle and diet for the first time. Starting from Keys’ studies, many other scientific researchers have analyzed the association between dietary habits and chronic diseases [3] or, more generally, the role of nutrition in individual and public health. It represents a very personal matter and requires understanding on the part of each individual as well as the society. In particular, dietary diversification programs need to take into account the existence of vulnerable (high risk) groups, like children, elderly people, malnourished, immunocompromised, acutely or chronically ill people and pregnant and/or lactating women, which have special nutritional needs [5]. Pregnancy, in particular, is a critical period of plasticity whereby fetal development may be significantly influenced by inherited genetic profile and various environmental factors, such as maternal hormones, living habits and nutrients which may have a strong potential to exert a long-lasting impact on the offspring’s growth and health into adulthood [6]. Maternal nutrition has caught much attention by governments and scientists and recently a high number of systematic reviews have examined effects of nutritional interventions in pregnancy on birth outcomes [5]. In this context, special attention has been paid to the finding that overall maternal diet may confer supplementary protection against of the neural tube defects (NTDs) and the mediterranean dietary pattern, in particular, seems to be associated with the highest risk reduction [7], as it represents an important source of methyl donors, like folate and vitamin B12, essential cofactors in several pathways of cellular processes implicated in placental and fetal growth and development [8].

Furthermore, many studies, report a relatively low occurrence of SB in countries where the Mediterranean diet is originally used, like in Southern Europe [9].

Paradoxically, despite these and many other evidences certifying this dietary pattern as one of the healthiest, we are nowadays assisting to the progressive abandonment of Mediterranean diet, mainly by young generations (including childbearing age women), in the most of mediterranean countries. A “nutritional transition process” is affecting the southern and eastern mediterranean countries where problems of under and malnutrition coexist with overweight, obesity and diet-related chronic diseases. According to data from 15,000 respondents analyzed by GfK-Eurisko market research organisation presented during a meeting about nutrition in Milan on May 2016, Italians are, one might say, “forgetting” the Mediterranean diet: in particular, just two in 10 Italians eat the four recommended daily portions of fruit, three in 10 eat enough vegetables, and one in three eats enough fish, revealing a trend towards more north american eating habits, [10] mainly represented by energy-dense diets comprised of high levels of refined grains, red meat, added sugars, and added fats as well as consuming foods from away-from-home sources like fast food and cafeterias [11]. Thus, the erosion of the Mediterranean diet heritage, by the progressive loss of its adherence among mediterranean basin populations, is alarming as it has undesirable and very important impacts not only on health but also on social life, economic laws and environmental trends [10].

### Folate, folic acid and neural tube defects

Birth defects are one of the leading causes of infant mortality worldwide and affect an estimated 1% to 3% of all births [12]. About 276,000 babies die within 4 weeks of birth every year worldwide from congenital anomalies (7% of all neonatal deaths), which represent structural or functional anomalies (e.g. metabolic disorders) that occur during intrauterine life and can be identified prenatally, at birth or later in life. Major anomalies (like NTDs) involve significant structural changes that have important medical, social or cosmetic consequences for the affected individual, commonly requiring medical and/or surgical intervention. NTDs are a relevant example of major congenital anomalies, as they represent one of the most common types of the congenital defects [13]. Development and closure of neural tube occur very early during normal embryonic development, between the 18th and 28th days after fertilization. Failure of the neural folds to fuse properly in the midline and form the neural tube in this delicate phase results in NTDs [14].

The term dysraphism is defined as “defective fusion”; thus, in this particular case, it refers to failure of normal midline fusion and indicates the existence of a persistent continuity between the posterior neural ectoderm and the cutaneous ectoderm. Cranial dysraphism, originating from failure of cranial portion of the neural tube to close, classically includes anencephaly and encephalocele, whereas spinal dysraphism (due to failure of caudal segment of neural tube closure) encompasses an heterogeneous group of congenital disorders involving the spine and spinal cord due to aberrant formation of the midline mesenchymal bony and neural structures [15]. Spina bifida (SB) is the term most commonly used for these dysraphic failure and the different subtypes of SB are based on the degree and the pattern of malformation associated with neuroectodermal involvement as well as of the entity of the herniation/exposure of the spinal cord or meninges [12, 16].

A practical clinical classification splits NTDs into two categories with distinct clinical management and prognosis: open NTDs, when neural tissue is exposed to the environment through a congenital skin defect, continuously or intermittently leaking cerebral spinal fluid (CSF), and closed NTDs, covered by skin and not leaking CSF [15, 16].

From an epidemiological point of view NTDs constitute a major health burden (0.5-2/1000 pregnancies worldwide), and still remain a preventable cause of still birth, neonatal, and infant death, or significant lifelong sequelae [15]. Every year, in Europe globally about 5000 fetuses are diagnosed with SB. The prevalence of NTDs shows large differences between the various countries; regional rates in Europe can be located within 25.7 per 10,000 births in Glasgow (United Kingdom) and 4.4 per 10,000 births in Emilia-Romagna (Italy). Up to now, the total prevalence at birth (live births + induced abortions + stillbirths) of NTDs in Italy is around 6 per 10,000 births, where a 50% is represented by SB cases, 40% by anencephaly and the remaining 10% by encephalocele (cases detected by the regional registries of Congenital malformations) (http://www.iss.it/cnmr/index.php?lang=1.&id=2474&tipo=77). The burden of illness is heavy for both the woman and her offspring, and in the case of SB, there are lifelong clinical and economic consequences [17]. Although SB has a lower case fatality rate than other neural tube defects (approximately 7%, compared with 46% for encephalocele, and 100% for anencephaly), it can result in severe life-long morbidity. This likely contributes to the high pregnancy termination rates following prenatal detection of SB [12].

The etiology of NTDs is still unknown but one of the possible reasons may be a disturbance of the one-carbon metabolism pathway [14]. A wide literature on possible association between NTDs and several maternal risk factors, including disadvantaged economic conditions, smoking habit, excessive caffeine intake, air pollution, low or high maternal age, body mass index (low or high), diabetes, antiepileptic drug therapy, polyhydramnios, is nowadays available, with often statistically inconclusive results, though [18]. In most cases, NTDs are considered to have a multifactorial origin [7].

Genetic predisposition and environmental influences are thought to contribute to NTDs. Evidence suggests that a mutation in MTHFR gene [19], an enzyme that catalyzes the conversion of 5, 10-methylenetetrahydrofolate (5, 10-MTHF) to 5-methyltetrahydrofolate (5-MTHF) and provides the methyl group needed for remethylation of homocysteine to produce methionine. The T allele of MTHFR 677C > T is associated with an increased risk of NTDs in the western population [14]. Folic acid consumption may help to decrease the effects of the low enzymatic activity of MTHFR [19].

During the past decades a determinant role of maternal nutrition, as an environmental factor, has been emphasised: low maternal dietary intakes of many nutrients, such as a vitamin B12, niacin, iron and magnesium have been found to be statistically associated with SB in the offspring, even though literature attention mainly centres on the rule of the B-vitamin folate [7]. Folate is a water-soluble B vitamin (B9) that is naturally present in wide variety of foods, in different concentrations. Beef liver, black eyed peas, yeast, asparagus, brussels sprouts, spinach, and other dark green leafy vegetables show the highest levels of folate, followed by other alimentary products like avocado, bananas, citrus fruits, nuts, eggs (egg yolk in particular), dairy products, seafood, and grains [20].

D.J. McKillop et al. showed that the retention of folate in various foods is highly dependent both on the food in question and the method of cooking: folates of animal origin, for example, were found to be relatively stable to cooking even for prolonged periods, while the folate availability of green vegetables was demonstrated to be less stable to heat and strictly connected to the method and duration of cooking [21].B9 is also available as supplemental form artificially added to food, through a process called fortification, and even as a dietary supplement. Technically, folate is the generic term for both naturally occurring food folate, mostly in polyglutamate form, and folic acid, the fully oxidized monoglutamate form of the vitamin used as dietary supplement and for the food fortification [20]. The latter is a synthetic compound and is metabolised differently to naturally occurring forms of folates: whereas food folates are hydrolysed to monoglutamates in the intestinal mucosa, absorbed by active transport across the intestinal mucosa and then transported (either in methyl or formyl forms) around the body via the circulatory system, folic acid, after entering the blood stream through both an active and passive transport, requires a two-step reduction, which occurs mainly in the liver, via dihydrofolate reductase (DHFR) to tetrahydrofolate (THF) to allow folic acid to be used for metabolic processes [22]. The bioavailability of folic acid is approximately 70% higher than that of natural folate, although there are wide variations mainly depending on the methodology used in the measurement [13]. Folate functions as a coenzyme or co-substrate in single-carbon transfers in the synthesis of nucleic acids (DNA and RNA) and metabolism of amino acids. One of the most important folate-dependent reactions is the conversion of homocysteine to methionine in the synthesis of S-adenosyl-methionine, an important methyl donor. Another folate-dependent reaction, the methylation of deoxyuridylate to thymidylate in the formation of DNA, is required for proper cell division. An impairment of this reaction initiates a process that can lead to megaloblastic anemia, one of the hallmarks of folate deficiency [20].

Nevertheless, most tissues, including the liver, have limited ability to reduce folic acid due to low activity of DHFR. It is also known that the process of absorption and biotransformation of folic acid to its active form (5-methyltetrahydrofolate) is saturated at doses in region of 200–400 μg of folic acid. The limitation of this metabolic process results in inability to metabolise high doses of folic acid, which leads to the appearance of unmetabolised folic acid (UFA) in the circulation [22]. The total body content of folate is estimated to be 10 to 30 mg, with about half of this amount stored in the liver and the remainder in blood and body tissues. Serum folate concentration is commonly used to assess folate status, with a value above 3 nanograms (ng)/mL indicating adequacy. This indicator, however, might not reflect long-term status as it is sensitive only to recent dietary intake. Thus, an other indicator, erythrocyte folate concentration (EFC), has been proposed to provide a longer-term measure of folate intakes and tissue folate stores [20]. The latest scientific evidence suggests that a EFC above 1000 nmol/l is the level required for optimal NTDs prevention. The latest dose-response studies report that in case of adoption of periconceptional FA supplementation as single strategy, a period of more than 12 weeks with 400 μg per day is needed to achieve the reported level of EFC; regarding the food fortification approach, on the contrary, literature data about the precise amount and duration of fertile women population’s exposure are not yet available.

Anyway, more kinetic studies are needed to define with greater certainty the duration of supplementation necessary to achieve optimal ECF to overthrow the risk of NTDs [17].

### Neural tube defects prevention: supplementation and/or food fortification?

The exact etiology of many birth defects remains frequently unknown despite the high prevalence, making primary prevention efforts very challenging. A notable exception to this is the evidence of a widespread decline in the prevalence of NTDs following the periconceptional (period including pregnacy’s first trimester) supplementation and mandatory fortification of grain products with folic acid in several countries [12].

In 1991, one RCT demonstrated for the first time that periconceptional supplementation of folic acid was able to prevent the recurrence of NTDs [23]. The next year, another RCT confirmed this finding, showing how a multiple micronutrient supplement containing folic acid was capable of lowering the occurrence of NTDs [24].

Basing on these and other findings, in the same year the US Public Health Service (USPHS) recommended that all women of childbearing age capable of becoming pregnant take 400 μg of folic acid per day [25]. Evidence that periconceptional folic acid supplementation can substantially decrease the prevalence of NTDs was further strengthened by a study conducted in China between 1993 and 1995 during a public health campaign among women preparing for marriage, affirmed that the risk of NTDs among the fetuses or infants decreased by between 40 and 85% for women taking folic acid supplement more than 80% of the time [26]. Nevertheless, many of the USPHS committee members were aware that the recommendation that women take supplements daily would be unlikely to prevent all folate-related NTDs for several reasons like poor compliance of women not planning a pregnancy, expensive tablets and difficulty in taking folic acid daily for a long period [25]. A very relevant issue that stands out in this context is the high rate of unplanned/unintended pregnancies and births. Technically, an unintended pregnancy is a pregnancy that is reported to have been either unwanted or mistimed (pregnancy occurred earlier than desired) at time of conception (https://www.cdc.gov/reproductivehealth/unintendedpregnancy/). Basing on recent literature data approximately half of pregnancies are estimated to be unplanned [25]. All over the world, both in developed and low income countries, women have more pregnancies and children than they expect and become pregnant much sooner than desired. The amount of time a woman typically spends avoiding unwanted or mistimed pregnancies, especially in the developed world, has increased in recent decades, due to a progressive urbanization process as well as social and economic development, leading many couples to want fewer children than in the past [27]. Wherever it occurs, unintended pregnancy is associated with an increased risk of problems for the mom and baby, as if a pregnancy is not planned before conception, a woman may not be in optimal health for childbearing (https://www.cdc.gov/reproductivehealth/unintendedpregnancy/). Because many women remained at risk for having children with folate-related NTDs, the United States government decided to institute mandatory food fortification with folic acid in 1996. All enriched breads, cereals, flours, cornmeals, pastas, rice, and other grain products were required to contain 140 μg of folic acid per 100 g of grain by January 1998.

As grains and cereals are extensively consumed in the United States, these products became very quickly important sources of folic acid intake for American people (especially for pregnant women, which usually avoid folate rich foods like raw green leafy vegetables being scared by toxoplasmosis). In this vein, also the Canadian government required the addition of folic acid to many grains, including white flour, enriched pasta, and cornmeal, since 1998, followed by other countries like Costa Rica, Chile, and South Africa. This strategy has been ultimately adopted by approximately 80 countries, with remarkable success. Data from many countries showed a significant decline in NTDs rates after mandatory fortification [20, 25].

In 2009, the US Preventive Services Task Force (USPSTF) recommended that “all women planning or capable of pregnancy take a daily supplement containing 400 to 800 μg of folic acid (A recommendation).” This recommendation differs from the previously reported USPHS recommendation in suggesting a range of folic acid (instead of a definite value) as the target dose [19]. Two recent studies examined whether taking daily supplements were associated with any kind of additional protection in the US population in the “fortification era” [28, 29]. Both of these projects showed that women who had infants affected by NTDs were no less likely to take supplements than women who had unaffected infants, suggesting that fortification of grain products, despite the current modest level, is preventing most, if not all, folate-related NTDs. Thus, the 400 to 800 μg per day recommended by the USPSTF may be more than is needed to prevent NTDs. Basing on these findings question should be asked of whether USPSTF recommendation should be rejected because fortified food is already providing sufficient folic acid to prevent NTDs. However, it should be taken in account that too little is still known about how folic acid prevents NTDs [25]. Furthermore, mandatory fortification has the important disadvantage that, unlike taking folic acid supplements, the entire population is “chronically” exposed, an issue that has caused and is still causing considerable debate in the European Union. Doubs have been expressed regarding the possibility that exposure to high doses of folic acid for an extended period might cause an increased risk of cancer, asthma, cognitive problems, twin pregnancy, and autism and may mask vitamin B12 deficiency anemia; however, up to now the data published in literature do not demonstrate any of these harms, with the exception of masking vitamin B12 deficiency anemia in elderly persons [30]. Thus, whereas many European countries have issued recommendations for folic acid supplementation for women of reproductive age, or specifically for those who intend to become pregnant, mandatory fortification programmes do not yet exist in Europe. Recently a european population based observational study, conducted by B. Khoshnood et al. [31] evaluated more than 11,000 cases of NTDs not associated with chromosomal abnormalities, basing on 28 national registers (including the italian regions Emilia-Romagna and Tuscany) included in the “European Surveillance of Congenital Anomalies Network” (EUROCAT; www.eurocat-network.eu). This study covered approximately 12.5 million births in 19 countries between 1991 and 2011. Overall, the pooled total prevalence of NTDs during the study period was 9.1 per 10.000 births. Prevalence of NTDs fluctuated slightly but without an obvious downward trend, with the pooled total prevalence of NTDs in 2011 resulted similar to the one reported in 1991. The authors concluded that in the absence of mandatory food fortification, the prevalence of NTDs has, in fact, not decreased in Europe despite long standing recommendations aimed at promoting periconceptional folic acid supplementation and the existence of voluntary folic acid fortification [18, 31]. Despite the prevalence of periconceptional supplementation implementation has increased slightly, the achieved levels are still unsatisfactory and well below the level required to obtain an equity in social health of 100%. Recent data report, for example, a prevalence comprised between the level of 14.8% registered in France in year 2010 [32], up to a 55.5% observed in the Netherlands (from 2009 to 2010) [33]. Italy finds itself almost halfway, with a level of 23,5% in 2012 [34]. Moreover, we noticed a wide dissimilarity in the formulation of the distinct recommendations between the different European countries, as well as a lack of clarity of the informative messages for the target population. A few studies conducted in Italy on female blood donors indicated a EFC in women of 15-44 years of 695 nmol/l (equivalent obtained by microbiological method) [35]. This value is similar to that of 686 nmol/l observed in the United States in 1988 to 1994 in women of 15-44 years during the pre-fortification era [36], thus consistently lower than the minimum level of 906 nmol/l recommended by the WHO for the general population [37]. Therefore, in the absence of food fortification, because the average time required to reach the erythrocyte folate concentration that maximizes the risk reduction with the only periconceptional folic acid supplementation may be as long as 40 weeks, the prescription should be made to all women of childbearing age, whereas, however, poor clarity of informative campaigns and the overall low compliance of young women should be taken into account [38].

## Conclusions

In Italy, like in the rest of Europe, an efficient prevention plan against NTDs does not exist yet. Basing on the experience gained on the other side of the ocean, although conclusive data about the real needs of folate during pregnancy are not available, the benefit in terms of clear reduction of NTDs incidence seem to be significantly higher than the risk associated with the assumption, for a long period, of an amount of folic acid exceeding the basic necessities. In Italy folic acid 400 μg/tab is provided free of charge by the NIH if requested by medical prescription. The 120 tablets pack is totally free in some regions whereas in others a minimum amount of money (prescription charge) has to be paid. If the percentage of births from unplanned pregnancy were judged acceptable the most reasonable choice may be the periconceptional prescription of folic acid 400 μg/day for all women (couples) starting to think about having a child or that do not categorically exclude that possibility. Since the rate of unintended/mistimed pregnancies seems to be still quite elevated and there is no available source reporting a statistically significative strong evidence of concrete risks in large scale chronic exposure to moderate amounts of folic acid, we believe that the introduction of a well established program of daily consumer foods fortified with folic acid (similar to the USA operative plan) combined with an efficient information and awareness campaign about the crucial importance of an healthy and balanced diet during and before pregnancy, addressed to families and, more specifically, to women of childbearing age, may, therefore, play, in Italy as in the other European countries, a key role in the fight against the drama of these disabling pathologies.

## Appendix

**Table 1:** NTDs prevention: take home messeges

NTDs	NTDs constitute a major health burden, responsible for about 0.5-2/1000 pregnancies worldwide and still representing a preventable cause of still birth, neonatal, and infant death, or significant lifelong sequelae.
Folate and folic acid sources	Folate is a water-soluble B vitamin naturally present in foods such as dark green leafy vegetables, legumes, and oranges. Folic acid is the synthetic form of folate. Folic acid is nowadays available as multivitamin, or single supplement and as essential nutrient used for cereal grain products fortification.
Folate and NTDs prevention	Women of childbearing age are at risk of having a pregnancy affected by NTDs, with variable rates from country to country. Convincing evidence shows that the fulfilment of adequate folate levels during the most critical periconceptional period (4 weeks before conception through the first trimester) can substantially decrease the prevalence of NTDs. In this context, maternal diet may play a key role in protection against these major congenital abnormalities and the Mediterranean dietary pattern seems to be associated with the highest risk reduction.
Mediterranean diet and Nutritional transition process	The Mediterranean dietary pattern represents an important source of methyl donors, like folate, essential cofactors in several pathways of cellular processes implicated in placental and fetal growth and development. Despite the evidences, we are nowadays assisting to the progressive abandonment of Mediterranean diet in favour of more unhealthy north American eating habits.
Prevention strategies	A balanced diet and a healthy life-style alone, despite the benefits, seem to be not enough for the prevention of NTDs. The main question is which intervention, between folic acid supplementation, foods fortification or both, linked to a healthy life-style and diet pattern may represent the best method in preventing NTDs. The relative failure of the periconceptional folic acid supplementation policy has led many countries to introduce mandatory fortification of flour with folic acid. Studies following fortification showed a significant decline in the NTDs rates in those countries.
Folate metabolism status assessment	There are no universally accepted cut-off points to define deficiency of folate. Erythrocyte folate concentration (EFC), is definitely the best available indicator to assess long-term measure of folate intakes and tissue folate stores. The latest scientific evidence suggests that a EFC above 1000 nmol/l is the level required for optimal NTDs prevention.
Chronical exposure to Folic acid supplementation: adverse effects?	Literature data report a possible association between long lasting high blood folate levels and an increased risk of many pathologic conditions like many types of cancer, asthma, cognitive problems and autism as well as the coverage of vitamin B12 deficiency anemia. Nevertheless, no clear consensus and concrete statistical evidence regarding the safety of folic acid has been met, due to discrepancies between results from different studies.
Conclusions	Basing on the experience gained in the US, the benefit in terms of clear reduction of NTDs incidence seem to be significantly higher than the risks associated with the assumption, for a long period, of an amount of folic acid exceeding the basic necessities. The introduction of a well established program of daily consumer foods fortified with folic acid combined with an efficient information and awareness campaign promoting a healthy and balanced diet before and during pregnancy may represent an important strategy to reduce the incidence of these pathologies.

## Abbreviations

EFC: Erythrocyte folate concentration
EUROCAT: European surveillance of congenital anomalies network
MTHFR: Methylene-tetra-hydro-folate-reductase
MUFA: Mono-Unsaturated long chain fatty acids
NTDs: Neural tube defects
PUFA: Poly-Unsaturated long chain fatty acids
RCT: Randomised controlled trial
SB: Spina Bifida
TAB: Tablet
UFA: Unmetabolized folic acid
USPSTF: US preventive services task force

## References

[1] Brambila-Macias J, Shankar B, Capacci S (2011). Policy interventions to promote healthy eating: a review of what works, what does not, and what is promising. Food Nutr Bull.

[2] Grosso G, Marventano S, D’Urso M (2016). The Mediterranean healthy eating, ageing, and lifestyle (MEAL) study: rationale and study design. Int J Food SciNutr.

[3] Altomare R, Cacciabaudo F, Damiano G (2013). The mediterranean diet: a history of health. Iran J Public Health.

[4] Davis C, Bryan J, Hodgson J (2015). Definition of the Mediterranean diet; a literature review. Nutrients.

[5] Black RE, Victora CG, Walker SP (2013). Maternal and child undernutrition and overweight in low-income and middle- income countries. Lancet.

[6] Geraghty AA, Lindsay KL, Alberdi G (2016). Nutrition during pregnancy impacts Offspring’s epigenetic status - evidence from human and animal studies. NutrMetab Insights.

[7] Vujkovic M, Steegers EA, Looman CW (2009). The maternal Mediterranean dietary pattern is associated with a reduced risk of spina bifida in the offspring. BJOG.

[8] Timmermans S, Steegers-Theunissen RP, Vujkovic M (2012). The Mediterranean diet and fetal size parameters: the generation R study. Br J Nutr.

[9] Botto LD, Lisi A, Bower C (2006). Trends of selected malformations in relation to folic acid recommendations and fortification: an international assessment. Birth Defects Res A Clin Mol Teratol.

[10] Dernini S, Berry EM, Serra-Majem L (2016). Med diet 4.0: the Mediterranean diet with four sustainable benefits. Public Health Nutr.

[11] Smith LP, Ng SW, Popkin BM (2013). Trends in US home food preparation and consumption: analysis of national nutrition surveys and time use studies from 1965-1966 to 2007-2008. Nutr J.

[12] Atta CA, Fiest KM, Frolkis AD (2016). Globale birth prevalence of Spina bifida by folic acid fortification status: a systematic review and meta-analysis. Am J Public Health.

[13] De-Regil LM, Peña-Rosas JP, Fernández-Gaxiola AC (2015). Effects and safety of periconceptional oral folate supplementation for preventing birthdefects. Cochrane Database Syst Rev.

[14] Li K, Wahlqvist ML, Li D (2016). Nutrition, one-carbon metabolism and neural tube defects: a review. Nutrients.

[15] Salih MA, Murshid WR, Seidahmed MZ (2014). Classification, clinical features and genetics of neural tube defects. Saudi Med J.

[16] JG MC (2015). A practical clinical classification of spinal neural tube defects. Childs NervSyst.

[17] Cawley S, Mullaney L, McKeating A (2016). A review of European guidelines on periconceptional folic acid supplementation. Eur J ClinNutr.

[18] Lanari M. Attuare un’efficace prevenzione dei Difetti del Tubo Neurale: si può, si deve. SIN informa. 2015. http://www.neonatologia.it/.

[19] Bibbins-Domingo K, Grossman DC, US Preventive Services Task Force (2017). Folic acid supplementation for the prevention of neural tube defects: US preventive services task force recommendation statement. JAMA.

[20] Centers for Disease Control and Prevention (2010). CDC grand rounds: additional opportunities to prevent neural tube defects with folic acid fortification. MMWR Morb Mortal Wkly Rep.

[21] McKillop DJ, Pentieva K, Daly D (2002). The effect of different cooking methods on folate retention in various foods that are amongst the major contributors to folate intake in the UK diet. Br J Nutr.

[22] Patel KR, Sobczyńska-Malefora A (2017). The adverse effects of an excessive folic acid intake. Eur J ClinNutr.

[23] Anonymous (1991). Prevention of neural tube defects: results of the Medical Research Council vitamin study; MRC vitamin study research group. Lancet.

[24] Czeizel AE, Dudas I (1992). Prevention of the first occurrence of neural-tube defects by periconceptional vitamin supplementation. NEJM.

[25] Mills JL (2017). Strategies for preventing Folate-related neural tube defects supplements, fortified foods, or both. JAMA.

[26] Berry RJ, Li Z, Erickson JD, Li S, Moore CA, Wang H (1999). Prevention of neural-tube defects with folic acid in China. China-U.S. collaborative project for neural tube defect prevention. NEJM.

[27] Sedgh G, Singh S, Hussain R (2014). Intended and unintended pregnancies worldwide in 2012 and recent trends. Stud Fam Plan.

[28] Ahrens K, Yazdy MM, Mitchell AA (2011). Folic acid intake and spina bifida in the era of dietary folic acid fortification. Epidemiology.

[29] Mosley BS, Cleves MA, Siega-Riz AM (2009). National Birth Defects Prevention Study. Neural tube defects and maternal folate intake among pregnancies conceived after folic acid fortification in the United States. Am J Epidemiol.

[30] Mills JL, Dimopoulos A (2015). Folic acid fortification for Europe?. BMJ.

[31] Khoshnood B, Loane M, de Walle H (2015). Long term trends in prevalence of neural tube defects in Europe: population based study. BMJ.

[32] Tort J, Lelong N, Prunet C (2013). Maternal and health care determinants of pre-conceptional use of folic acid supplementation in France: results from the 2010 National Perinatal Survey. BJOG.

[33] Manniën J, De Jonge A, Cornel MC (2013). Factors associated with not using folic acid supplements pre-conceptionally. Public Health Nutr.

[34] Mastroiacovo P, Nilsen RM, Leoncini E (2014). Prevalence of maternal preconception risk factors: an Italian multicenter survey. Ital J Pediatr.

[35] Zappacosta B, Persichilli S, Iacoviello L (2013). Folate, vitamin B12 and homocysteine status in an Italian blood donor population. NutrMetabCardiovasc Dis.

[36] Pfeiffer CM, Hughes JP, Lacher DA (2012). Estimation of trends in serum and RBC folate in the U.S. population from pre- to post-fortification using assay-adjusted data from the NHANES 1988-2010. J Nutr.

[37] Cordero AM, Crider KS, Rogers LM, Cannon MJ, Berry RJ (2015). Optimal serum and red blood cell folate concentrations in women of reproductive age for prevention of neural tube defects: World Health Organization guidelines. MMWR Morb Mortal Wkly Rep.

[38] Mastroiacovo P, Corchia C (2016). Riflessioni sulla prevenzione primaria dei difetti del tubo neurale in Italia e spunti per una raccomandazione basata sulle evidenze più recenti. Quaderni acp.

